# A Split-Plot Experimentation Strategy for Making Causal Inferences in Advanced Materials: Auxetic Polyurethane Foam Manufacturing and Processing Analysis

**DOI:** 10.3390/ma17133280

**Published:** 2024-07-03

**Authors:** Matthew S. Wadsworth, Md Jahan Deloyer, Omer Arda Vanli, Changchun Zeng

**Affiliations:** Department of Industrial and Manufacturing Engineering, FAMU-FSU College of Engineering, High Performance Materials Institute, Florida State University, 2525 Pottsdamer St., Tallahassee, FL 32310, USAoavanli@eng.famu.fsu.edu (O.A.V.)

**Keywords:** advanced materials, design of experiments, split-plot design, auxetic foam

## Abstract

Development of advanced materials is often time consuming and expensive because of the large number of variables involved and experiments needed. An effective experimentation strategy would accelerate development by reducing the required amount of experiments without sacrificing the obtainable information. In this paper, the development of auxetic polyurethane (PU) foams was discussed as a case study. Auxetic materials are materials with a negative Poisson’s ratio and have potential in many structural and functional applications. Auxetic PU foams are the most studied auxetic materials, and their manufacturing and properties are affected by many processing and environmental factors. This paper introduces a sophisticated design of experimental methodology to help reduce the experimental effort while effectively screening these factors. This methodology is then applied in an experiment to illustrate its utility and distinct advantages that greatly facilitate material development.

## 1. Introduction

Randomized controlled experiments are the gold standard for making causal inferences about factor effects [1]. Their construction, which requires complete randomization of treatment combinations to experimental units, is often difficult in agricultural, industrial, and research settings due to cost constraints. These cost constraints are likely to be encountered when there are factors with levels that are time consuming, expensive, or hard to change or hard to set. In split-plot designs [2], which originate from agricultural experimentation, the experimental units are first subdivided into relatively large portions, known as whole plots, and each of the possible levels of the hard-to-change factors (whole plot factors) are then randomly assigned to these plots. Whole plots are further divided into smaller portions, known as subplots, to which the levels of the easy-to-change (subplot) factors are randomly applied. Split-plot designs, which utilize this restriction in randomization, provide a significant cost-saving and cost-effective method for designing experiments in such settings.

Material scientists working with advanced materials often encounter experiments in which a single run is extremely costly and/or time consuming and using a completely randomized factorial design (CRD) is not realistic because it would be both time and cost prohibitive. A material is generally considered an advanced material if the material exhibits improvements in chemical, physical, or chemical and physical properties compared to the traditional material [3,4]. Development and adoption of these materials is often challenging and/or expensive. For example, there is significant interest in the development of lithium-ion batteries with higher energy densities for use in electric vehicles and energy storage. As part of the final stages of manufacturing, batteries are subjected to aging and formation stages. The length of time that a battery stays at these stages can range from several days to weeks [5]. The long formation and aging process, in combination with many other qualities of interest, including battery chemistry, material rheology, and lifecycle performance (i.e., calendar aging and cycle aging), lead to experiments with completion times measured in years [6,7,8]. Other advanced material fields in which the split-plot design could help to drive innovation, including auxetic materials, composites, and nanomaterials, where there are many process variables, long experimental times, and noisy factors.

In this study, auxetic foam is used as a case study to demonstrate the use of the split plot design for advanced materials research. An auxetic material is a material with a negative Poisson’s ratio. Most materials have a positive Poisson’s ratio, i.e., v>0. When a material with a positive Poisson’s ratio is stretched, the cross-section becomes thinner, and when compressed, it expands laterally. The cross-section of an auxetic material, on the other hand, expands laterally when stretched and contracts under compression. To many, this behavior is counterintuitive, but the classical theory of elasticity provides theoretical justification for the existence of materials with Poisson’s ratio values between −1≤v≤0.5 when the material is isotropic [9].

Materials with a negative Poisson’s ratio were first reported by Love, who discovered the auxetic properties of a single crystal pyrite [10]. Since then, other auxetic materials have been found to exist in nature, such as arsenic, cadmium, cubic elemental metals, a-cristobalite, and numerous biological tissues [11,12,13,14]. Research on auxetic materials began gathering momentum when isotropic auxetic foams were manufactured first by Lakes [15]. His discovery catalyzed research in auxetic materials, which led to the fabrication of a variety of artificial auxetic materials and structures, such as different polymers, metals, composites, ceramics, and fibers [16].

Auxetic polyurethane (APU) foam is one of the most widely studied materials in the auxetic materials field. The unique properties of auxetic PU foam include increased sound and energy adsorption, enhanced shear and indentation resistance, variable permeability, vibration transmissibility, and the ability to produce a synclastic curvature [17]. Some of the applications of auxetic PU foam include shock and energy absorption for athletics or prosthetics, smart filtration devices, drug delivery systems, and tissue scaffolds [18,19,20,21]. Although APU has been studied for a long time and different applications have been demonstrated, a firm understanding of the manufacturing/processing and properties relationship is lacking, resulting in difficulties in achieving consistency in product structure and properties.

In this study, we investigated APU process analysis as a case study to illustrate a split-plot experimentation strategy for making causal inferences in advanced materials. The importance of the processing temperature, heating time, volumetric compression ratio, and their interactions for the manufacture of auxetic foams is investigated. A split-plot experimental design was constructed, and samples were manufactured according to the order specified. Samples were tested and the significance of each of the processing parameters and their interactions were evaluated. A linear model was built using the data collected and its predictions were compared to several validation runs. The advantages of the split plot over other experimental design methods used in the auxetic foam literature are discussed.

## 2. Materials and Methods

### 2.1. Experimental Design and Analysis

A split-plot design of experiments (DOE) approach was used to investigate the effects of processing temperature, heating time, compression ratio, and their interactions. Processing temperatures of 140 and 200 °C, heating times of 12 and 18 min, and compression ratios of 2 and 10 were selected as the factor levels. A split plot design was generated using Design-Expert (Design-Expert^®^ v6, Stat-Ease^®^, Minneapolis, MN, USA) and statistical analysis was performed in both Microsoft Excel (Microsoft 365 Excel© v2405, Microsoft Corporation, Redmond, WA, USA) and R language (R© v4.4.0, Vienna, Austria). In Microsoft Excel, the equations for the degrees of freedom, sum of squares, mean squares, and F values of the ANOVA table were input into the spreadsheet manually. The *p*-values were determined using the F.DIST.RT function in Microsoft Excel. The ANOVA table calculations from Microsoft Excel were verified in R language using the aov function from the base stats package [22]. A linear mixed model was fitted using the lmer function from the lme4 library [23]. ANOVA tables for the mixed model were generated using the anova.lme function from the lmerTest library [24] and validated by manual calculation. Interaction plots were generated using the Effect function from the effects library [25]. Contour plots of the mixed model were generated using the plotLMER3d function from the LMERConvenienceFunctions library [26]. The experimental designs shown in Table 1 were generated using SAS JMP (JMP© v16.0.0, SAS Institute, Cary, NC, USA).

### 2.2. Materials and Fabrication

**Figure 1:**
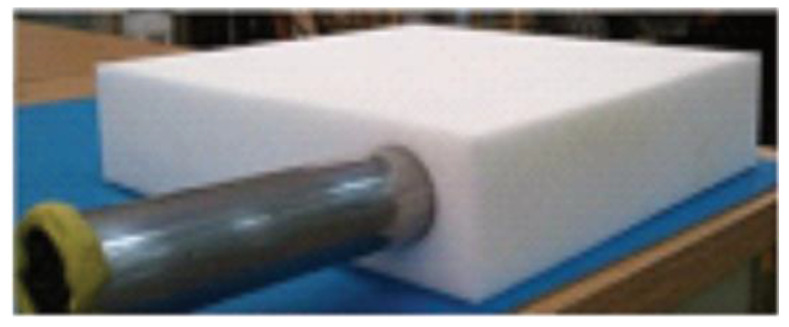
Photograph of raw polyurethane foam used for this study. A die cutter was used to cut cylindrical specimens for auxetic fabrication.

The raw material used was a white polyurethane (PU) foam supplied by McMaster-Carr (Elmhurst, IL, USA). The foam had a firmness of 0.7 psi (25% deflection) and a density of 44 kg m^−3^. It is a flexible foam with an open cellular morphology. Figure 1 shows a photograph of the foam. Conventional foam samples were converted into auxetic foams using a thermo-mechanical conversion process similar to the one described in [27]. Conventional foams with initial diameters of 23 mm, 36 mm, and 43 mm and lengths of 75 mm, 109 mm, and 129 mm were inserted into tube molds and compressed to a diameter of 20 mm and length of 60 mm, representing compression ratios of 2, 6, and 10 respectively. For conversion, two molds were placed in a forced convection oven at a processing temperature and heated for the amount of time specified by the DOE. After the desired heating time had elapsed, the molds were removed from the oven and allowed to cool to room temperature. Once cooled, foam samples were removed from the mold and stretched to prevent sticking. Samples were stored for at least one day prior to testing.

### 2.3. Poisson’s Ratio Calculation

Tensile tests were performed on auxetic foam specimens with an overall length of 75 mm and gauge length of 40 mm (Shimadzu AGS-J, Shimadzu, Kyoto, Japan) using a strain of 0.0025 s^−1^). Videos of the tensile deformation were captured using a video extensometer system (Shimadzu DV-201) and converted into static images at specified time intervals via MATLAB 2019b. A MATLAB routine was developed to automate the calculation of the length and width of the specimen for all generated images. Calculated length and diameter measurements were then used to calculate the transverse strain (*ε_x_*) and longitudinal strain (*ε_y_*) using the following equations:

(1) $$\varepsilon_{x}=\frac{\Delta l}{l_{0}}$$

(2) $$\varepsilon_{y}=\frac{\Delta d}{d_{0}}$$

where Δl is the change in length, $l_{0}$ is the original length, Δd is the change in diameter, and $d_{0}$ is the original diameter. The Poisson’s ratio was determined via linear regression to fit the strain–strain curve.

## 3. Results and Discussion

### 3.1. Fabrication of Auxetic PU Foams, Factor Selection, and Factor Levels

The distinguishing feature of an auxetic foam is its re-entrant cell structure [11]. A conventional piece of foam, however, has an outward cell structure or, in the idealized model, a convex shape. To convert the outward cell structure of a conventional foam to the re-entrant cell structure of an auxetic foam, a conventional foam is compressed and locked in the compressed state. Compression of the conventional foam bends and twists the ribs that make up the cells of the foam forming the re-entrant structure. Heating the compressed foam to its softening temperature relaxes the stress in the deformed ribs that would normally cause the compressed foam to expand back to its original geometry. Cooling the heated compressed foam to room temperature sets the foam in the compressed state. Using the appropriate processing temperature, heating time, and compression ratio for a conventional foam, a conventional foam can be converted into an auxetic foam.

There are two temperatures that are important for auxetic foam conversion, both of which vary widely [15,27,28,29,30,31,32,33,34,35,36]. In their work, Chan and Evans established what they called softening and conversion temperatures [28]. The softening temperature is the temperature at which the foam begins to experience deformation under heating (~200 °C). This was the temperature to which they set their oven for processing. However, heating the compressed foams to too high of a temperature could result in unwanted cell rib adhesion. Therefore, during conversion, foams were heated to a lower temperature, which they called the conversion temperature. It was recommended that the conversion temperature be 5–20 °C below the softening temperature to maximize relaxation and minimize adhesion. This conversion temperature acts as an upper temperature boundary for the conversion of conventional foams to auxetic foams while the glass transition temperature of the polyurethane is a rigid lower temperature boundary [32]. Many studies fabricate auxetic foams at temperatures toward the upper end of this region (≥160 °C) [28,30,36]; however, some have produced auxetic foams at lower temperatures using higher volumetric compression ratios (>5) [33] or extended heating times (>1 h) [36].

The time required for the sample to reach the conversion temperature is called the heating time. The heating times used can vary anywhere from 12 min up to 3 h and are dependent on the processing temperature used [30,33] and material composition [37]. One of the many challenges associated with auxetic foams is foam stability, both in the homogeneity of mechanical properties and long-term auxetic state stability [27,28,33,34,38,39]. The time-temperature profile is known to influence the stability of foams. Chan and Evans found that, after a short heating time, the foam was not set and the structure would revert back to its original size shortly after removal from the mold [28]. However, they did not quantify the length of time required for the properties to revert. Bianchi et al. found that, without an optimized time–temperature profile, foams would have a conventional Poisson’s ratio or would lose their auxeticity after several hours [33]. It has also been suggested that using an insufficient heating time can lead to the formation of three distinct regions in the foam: a conventional region, a weakened auxetic region, and an auxetic region [34]. It was theorized that foam cells in the weakened auxetic region could revert to a convention state under tensile stimulation and lead to the measurement of a positive Poisson’s ratio. The remaining conventional cell structure found within an auxetic sample may also explain issues with long-term stability [34]. Further, eliminating the conventional and weakened auxetic regions using a sufficient heating time could also help to reduce or eliminate the inhomogeneities of auxetic foam samples.

The largest auxetic foam study to date found that the volumetric compression ratio was the most important variable affecting the negative Poisson’s ratio [33]. The range of volumetric compression ratios can be determined using the stress–strain curve in compression for each of the three dimensions [28]. The stress–strain curve in compression for a conventional foam has three characteristic regions, which occur in sequence: a linear elastic region, a Plateau region, and a region of rapid densification. In the plateau region, the cell ribs of the conventional foam bend and buckle, forming the auxetic cells. Setting the foam in the compressed state anywhere in the plateau region for all three dimensions will produce an auxetic foam. Volumetric compression ratios used in literature vary with ratios as small as 2–3 up to ~20 able to produce auxetic foams [28,33].

Processing temperature, heating time, and volumetric compression ratio are the three controllable variables most commonly used or manipulated to investigate the properties of auxetic foams. The discussion in the preceding paragraphs discussed their importance and the wide range of settings that can be used to manufacture auxetic foams. Some of the reasons for the wide processing range may be attributable to other factors, but even within foams with identical compositions, a wide range of processing conditions can be used to make auxetic foams with identical or nearly identical Poisson’s ratios [30]. Constructing a mathematical model over most of the known experimental region could help to investigate, accelerate, improve, and better exploit the auxetic foam manufacturing process. For this study, processing temperatures of 140 and 200 °C, heating times of 12 and 18 min, and volumetric compression ratios of 2 and 10 are used. They encompass majority of the conditions for auxetic PU foam manufacturing reported in the literature. These values are used as the high and low levels for the DOE and allow for the construction of a mathematical model over the most widely investigated experimental region for auxetic foams.

### 3.2. Experimental Design and Design Execution

Many scientists and engineers are familiar with CRD. It is simple to execute, and all of the factor effects and errors can be estimated. However, many process variables, sharing of equipment, equipment cost, and experiment replication can quickly increase the amount of experimental time and resources required to complete an experiment, making CRD infeasible. In these situations, it may be more efficient to conduct experiments in groups or batches. Batched experimentation is already fairly common in auxetic foam research, with [27,31,32,33,37,38] using molds with multiple metallic tubes to produce samples using the same oven conditions but different volumetric compression ratios. Using an experimental design that allows for “batching” of experimental runs would enable a more in-depth analysis of the data already being collected. One such design is the split plot.

The split-plot design differs from that of the traditional completely randomized design in several ways. In the completely randomized design, it is possible for every factor setting to be changed between runs. However, in the split-plot design, the settings of some factors are changed more frequently than others. For example, Table 1 shows the auxetic foam experiment arranged as if it were to be conducted as a completely randomized experiment and Table 2 shows the split-plot design used for this study. In Table 1, every row has a new combination of high and low factor levels, as shown in the pattern column, and 16 individual experiments or oven trials are required to make 16 samples. In Table 2, the first two factors, processing temperature and heating time, are considered hard-to-change and only change every other row. These groupings of hard-to-change factors are referred to as whole plots. The last factor, the compression ratio, is considered easy-to-change and its setting is randomly assigned for every row within a whole plot. The reduction in the number of randomizations in the split-plot design is referred to as a restriction in randomization and is used to reduce the number of times the settings of difficult or hard-to-change factors must be altered. In the case of our auxetic foam experiment, executing the split-plot design cuts the number of oven trials in half to 8 while still producing the same number of samples.

**Figure 2:**
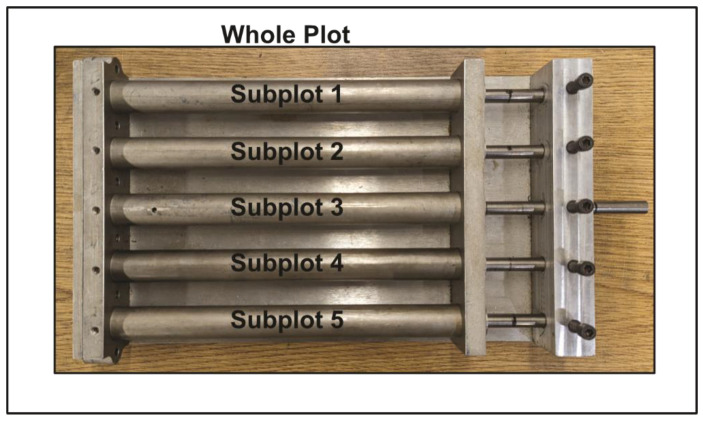
Illustration of the auxetic foam experimental setup with the mold used to manufacture the auxetic foam samples.

Split-plot designs can be executed in one of two ways. In the first approach, a whole-plot treatment is applied to a group of samples undergoing different subplot treatments. This approach is common in agriculture where it is easier to apply some experimental factors, such as plant variety, to a whole field or large groupings within the field (the whole plot), while others, such as fertilizer, can be changed within the field or within a large group of samples (the subplot) [40,41]. In the second approach, a whole-plot treatment is applied to a lot or group of samples, which can then be subdivided for treatment with the subplot factors. This approach is more likely to be found in an industrial setting [42]. In this study, the former approach was used. Using observational units one and two from Table 2 as an example, two auxetic foam samples were loaded into separate tubes on the mold, labeled subplot 1 and subplot 2 in Figure 2. The plungers were then set to the appropriate setting for the volumetric compression ratio specified by the DOE. In this case, the subplot 1 plunger was set to a compression ratio of 10 and the subplot 2 plunger was set to a compression ratio of 2. The mold, that is, the whole plot, was then placed in the oven heated to 140 °C for 12 min. This process was repeated 8 more times until the DOE was completed to produce 16 observational units or samples.

### 3.3. Evaluation of Experimental Design

**Figure 3:**
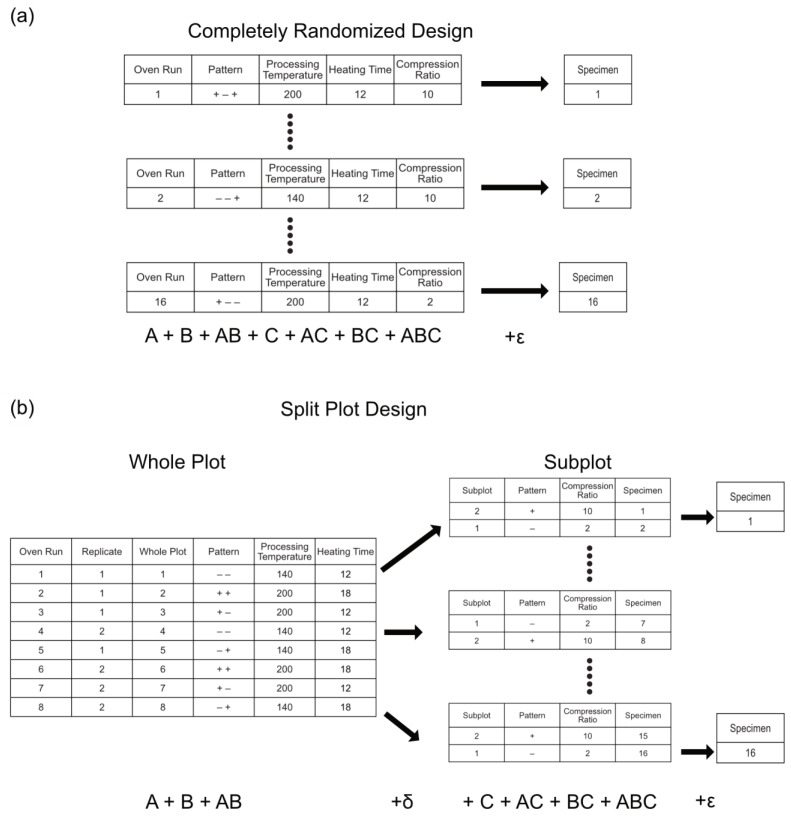
Schematics showing: (**a**) CRD and the respective linear model, and (**b**) the split-plot design and respective linear model.

Like CRD, replicated split-plot models can be analyzed using either normal probability plots or analysis of variance (ANOVA). However, because the subplot factor levels are changed more frequently than the whole-plot factor levels, the split-plot linear model has two different error terms or error strata, one for the whole plot (δ) and one for the subplot (*ε*), to account for the different randomizations. As a result, split-plot designs usually cannot be analyzed as if they were CRD, which only has a single error term (*ε*). This is illustrated in Figure 3, which shows the location of the error terms in CRD (Figure 3a) and split-plot models (Figure 3b). When using normal or half-normal probability plots, the effects for each error strata are plotted on separate normal probability plots. The assignment of effects to probability plots is not straightforward at first glance. A method for the assignment of effects to the whole-plot strata and subplot strata was introduced by Bisgaard to aid in classification [43]. Generally though, the whole-plot main effects and their interactions are assigned to the whole-plot normal probability plot. The subplot main effects and their interactions are assigned to the subplot normal probability plot. The interactions between the whole-plot and subplot effects are assigned to the subplot normal probability plot. Analysis of all effects on a single normal probability plot can result in disregarding or misinterpreting significant effects [43].

Different variations of the split-plot design have been presented in literature, each having a different ANOVA table [44]. The ANOVA table for the design used in this study is shown in Table 3. The calculation of the sum of squares, degrees of freedom, and mean squares for the main effects and interactions are the same as in completely randomized design. The appropriate error terms for the hypothesis tests are derived from the expected mean squares of the whole plots and subplots. In this design, the appropriate error terms are $\sigma_{sp}^{2}+c\sigma_{wp}^{2}$ for the whole plot and $\sigma_{sp}^{2}$ for the subplot. Statistical analysis of the design was completed via spreadsheet calculation. The sum of squares was calculated using the equations shown in column 4 of Table 3, where *i*, *j*, and *l* represent the levels of processing temperature, heating time, and compression ratio, respectively, and *k* represents the number of replicates. A *p*-value of 0.05 was used to test for significant factors and is indicated by * in Table 4. The results in Table 4 indicate that all of the main effects are highly significant, which is consistent with the literature [33,45,46]. The two-factor interactions but not three-factor interactions were also found to be highly significant. This indicates that the main effects and their two-factor interactions are very important to the manufacture of auxetic foams.

### 3.4. Model Construction and Validation

In addition to analyzing significant effects, an investigator may want to fit a regression equation to estimate the response based on the processing conditions. In completely randomized design, regression models are fit using ordinary least squares (OLS) regression. As discussed above, the restricted randomization in the split plot introduces a second error term and, as a result, split-plot designs cannot, with a few exceptions, be modeled with OLS [43,47]. Instead, split-plot models are typically fit with generalized least squares (GLS) or mixed models to account for the two error terms. Here, the data were fit with a linear mixed model. A linear mixed model incorporates both fixed effects, which model the effects of the factors, and random effects, which model the effects of the whole plots and subplots. The fixed effect regression coefficients for the reduced model, i.e., a model only incorporating the regression parameters for the significant effects from Table 4, and their 95% confidence intervals are shown in Table 5.

**Figure 4:**
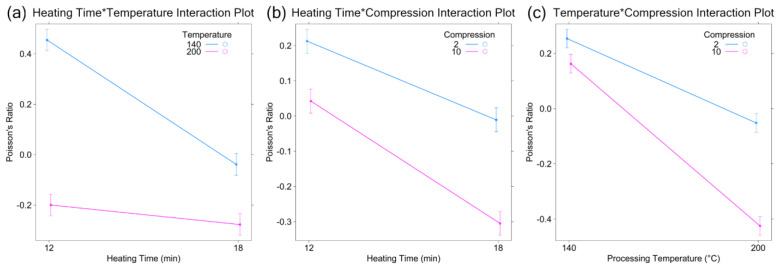
Interaction plots generated from the linear mixed model for: (**a**) heating time and temperature, (**b**) heating time and compression ratio, and (**c**) processing temperature and compression ratio.

**Figure 5:**
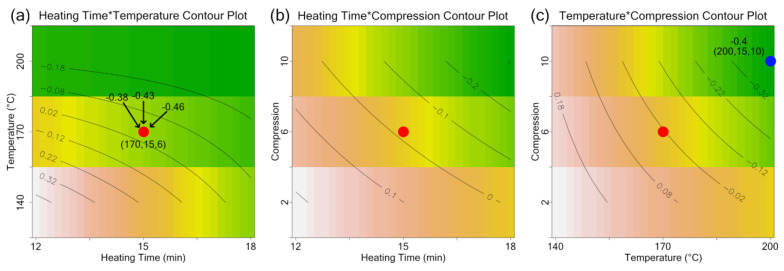
Contour plots generated using the linear mixed model for: (**a**) time and temperature, with a red dot indicating the location of a validation run; (**b**) time and compression; and (**c**) temperature and compression, with a blue dot indicating a validation run. The red dot represents the same experimental point across all three plots but plotted using different process variables as the axes.

The interaction plots and contour plots generated using the mixed model, shown in Figure 4 and Figure 5, suggest that reduction of the Poisson’s ratio can be achieved by increasing the processing temperature, heating time, and compression ratio. The interaction plot is a visualization tool for assessing the relative size of the main effects and interactions. An interaction plot of any two factors is constructed by connecting the average response at the two levels of the first factor (for the same level of the second factor) with a straight line. This straight line reflects the linear relation assumption and further aids in assessing the size of the interaction based on the slopes of the two lines. Figure 4a, Figure 4b, and Figure 4c show, respectively, that AB (Temperature–Time), BC (Time–Compression), and AC (Temperature–Compression) interactions are highly significant because the two lines in each figure have different slopes. Several validation points were run at the center point and edge of the experimental region to check the validity of the model. The Poisson’s ratios for points at the edge of the processing temperature and time regions (Figure 5c, blue) were found to be approximately the same as the value predicted by the model; however, the Poisson’s ratios for the center points (Figure 5a,b, red) were found to have a significantly lower Poisson’s ratio than that predicted by the model. This suggests that there may be a curvature in the experimental region or, alternatively, that the relationship between processing temperature, heating time, and compression ratio is not linear. This hypothesis aligns well with data presented in references [30,33,36]; however, a more formal test of curvature is needed to confirm the hypothesis. Significant variability in the Poisson’s ratio for multiple specimens was also observed, suggesting that there may be variability somewhere in the process (i.e., foam manufacturing, auxetic conversion, Poisson’s ratio measurement, etc.).

### 3.5. Advantage of Using Split-Plot Designs

The split-plot design offers several advantages over other experimental design methods. The first being the significant reduction in the number of trials that need to be executed, which was discussed in Section 3.2. Another example is illustrated here with a redesign of an experiment conducted by Bianchi et al., who determined the compression ratio to be the most significant factor for auxetic foam conversion [32,33]. In their experiment, 16 batches and a total of 80 auxetic foam samples were made using two temperature and time combinations, two cooling methods, and two initial foam diameters. Two different volumetric compression ratio ranges were used for each initial foam diameter, but they did overlap slightly. If the imposed dimensions are standardized to a diameter of 19 mm and length of 60 mm and include the radial and axial compression as separate factors, an alternative split-plot experiment can be obtained, shown in Table 6 (abbrev.), which produces 32 samples in 8 oven trials. Using this design would impose volumetric compression ratios of 2.92, 7.47, 7.47, and 19.14. This would allow for overlap to reproduce the same plots in the original experiment, although with fewer samples per cluster, and for statistical analysis of all the main effects and two-factor interactions to provide additional information. If the number of oven runs is increased to 16, a statistical model could be built to approximate the process, which could be augmented to gain additional insight into the design space.

The use of experimental designs for the development of auxetic foams appears to be limited. The two examples that were found used a Plackett–Burman design [46] and a Taguchi design (orthogonal array) [45], respectively. Both designs require significantly longer experimentation and development time than that required by split-plot designs. In addition, the Plackett–Burman design has the further shortcoming that the estimates of the effects of process variables are partially aliased (confounded) with the effects of two-factor interactions between other variables in the process [48]. This is problematic because the effects of the process variables or their interactions cannot be clearly estimated. Taguchi designs also have a similar limitation in that they are able to estimate interactions, but only if the investigator already knows they exist (e.g., proper assignment of factors to the appropriate columns in order to estimate their interaction) [49,50]. We were unable to find any literature that provided statistical analysis of interactions in the auxetic foam manufacturing process that would suggest which interactions should be included or excluded from an experimental design. Further, according to Minitab (Minitab© v21.4.2, Minitab LLC, State College, PA, USA), the design presented in [45] was not sufficient to estimate the effects of interactions between the variables in their study (temperature, time, and pressure). Both findings make the Taguchi design a surprising design choice given the limited understanding of the relationships between variables across the auxetic foam manufacturing process (i.e., composition, mixing, foaming, etc.) and the many considerations that must go into choosing an appropriate Taguchi array (aliasing, number of runs, etc.). The split-plot design, on the other hand, is significantly more flexible and can provide unaliased effects estimates for both the main effects and their interactions.

Lastly, split-plot designs are suitable for handling noisy processes. As shown in Figure 5a and elsewhere [39], there are variations or inhomogeneities in the properties of auxetic foams. Identifying factors contributing to these variations and optimizing the remaining factors could help to improve property uniformity. Taguchi designs are also used for optimizing processes with noisy factors; however, given that they disregard interactions and a variety of other criticisms [49], such as lack of randomization and method for choosing the optimal solution, it may be more appropriate to select or use alternative designs [51]. Box and Jones proposed the use of the split-plot design for noisy factors such as those related to the environment [42]. They showed that the process factors and the interactions between process factors and noisy factors can be more precisely estimated with the split plot than other viable alternative designs.

## 4. Conclusions

In this study, split-design methodology was used to construct experiments for the investigation of the effects of processing temperature, heating time, and volumetric compression ratio on the conversion of conventional foams to auxetic foams. It was found that all processing parameters and the interactions between these parameters were significant for the manufacture of auxetic foams. A linear mixed model was developed and validated at the edge of the processing temperature and time regions. Split-plot designs help to reduce the required number of experimental trials and can provide unaliased effects estimates for both the main effects and their interactions. The methodology has great potential to accelerate research and development in a variety of areas where experimentation involves multiple parameters.

## Figures and Tables

**Table 1:** Auxetic foam experiment arranged as a completely randomized design.

Observational Unit	Oven Run	Pattern	Processing Temperature	Heating Time	Compression Ratio
1	1	+ − +	200	12	10
2	2	− − +	140	12	10
3	3	− + −	140	18	2
4	4	− + +	140	18	10
5	5	+ − +	200	12	10
6	6	+ + −	200	18	2
7	7	+ − −	200	12	2
8	8	− − −	140	12	2
9	9	+ + −	200	18	2
10	10	+ + +	200	18	10
11	11	− − +	140	12	10
12	12	− + −	140	18	2
13	13	+ + +	200	18	10
14	14	− + +	140	18	10
15	15	− − −	140	12	2
16	16	+ − −	200	12	2

**Table 2:** Auxetic foam experiment arranged as a split plot.

Observational Unit	Oven Run	Whole Plot	Pattern	Processing Temperature	Heating Time	Compression Ratio
1	1	1	− − +	140	12	10
2	1	1	− − −	140	12	2
3	2	2	+ + +	200	18	10
4	2	2	+ + −	200	18	2
5	3	3	+ − −	200	12	2
6	3	3	+ − +	200	12	10
7	4	4	− − −	140	12	2
8	4	4	− − +	140	12	10
9	5	5	− + −	140	18	2
10	5	5	− + +	140	18	10
11	6	6	+ + +	200	18	10
12	6	6	+ + −	200	18	2
13	7	7	+ − +	200	12	10
14	7	7	+ − −	200	12	2
15	8	8	− + +	140	18	10
16	8	8	− + −	140	18	2

**Table 3:** ANOVA Table for split-plot design.

Source of Variation	Degrees of Freedom (DF)	Expected Mean Square (EMS)	Sum of Squares (SS)	Mean Squares (MS)	F-Statistic (F_0_)
Temperature (A)	a − 1	$\sigma_{sp}^{2}+{c\sigma }_{wp}^{2}+\frac{rbc\sum {\left(A\right)}_{j}^{2}}{\left(a-1\right)}$	$jkl\sum_{i}{\left({\bar{y}}_{i\ldots }-{\bar{y}}_{\ldots .}\right)}^{2}$	$\frac{{SS}_{A}}{{DF}_{A}}$	$\frac{{MS}_{A}}{{MS}_{ErWP}}$
Heating Time (B)	b − 1	$\sigma_{sp}^{2}+{c\sigma }_{wp}^{2}+\frac{rac\sum {\left(B\right)}_{k}^{2}}{\left(b-1\right)}$	$ikl\sum_{j}{\left({\bar{y}}_{.j..}-{\bar{y}}_{\ldots .}\right)}^{2}$	$\frac{{SS}_{B}}{{DF}_{B}}$	$\frac{{MS}_{B}}{{MS}_{ErWP}}$
AB	(a − 1)(b − 1)	$\sigma_{sp}^{2}+{c\sigma }_{wp}^{2}+\frac{rc\sum {\left(AB\right)}_{jk}^{2}}{\left(a-1)(b-1\right)}$	$kl\sum_{i,j}{\left({\bar{y}}_{ij..}-{\bar{y}}_{i\ldots }-{\bar{y}}_{.j..}+{\bar{y}}_{\ldots .}\right)}^{2}$	$\frac{{SS}_{AB}}{{DF}_{AB}}$	$\frac{{MS}_{AB}}{{MS}_{ErWP}}$
Error_WP_	ab(r − 1)	$\sigma_{sp}^{2}+{c\sigma }_{wp}^{2}$	$l\sum_{i,j,k}{\left({\bar{y}}_{ijk}-{\bar{y}}_{ij}\right)}^{2}$	$\frac{{SSE}_{WP}}{{DF}_{ErWP}}$	
Compression Ratio (C)	c − 1	$\sigma_{sp}^{2}+\frac{rab\sum {\left(c\right)}_{l}^{2}}{\left(c-1\right)}$	$ijk\sum_{l}{\left({\bar{y}}_{\ldots k}-{\bar{y}}_{\ldots .}\right)}^{2}$	$\frac{{SS}_{C}}{{DF}_{C}}$	$\frac{{MS}_{C}}{{MS}_{ErSP}}$
AC	(a − 1)(c − 1)	$\sigma_{sp}^{2}+\frac{rb\sum {\left(AC\right)}_{jl}^{2}}{\left(a-1\right)(c-1)}$	$jk\sum_{i,l}{\left({\bar{y}}_{i..l}-{\bar{y}}_{i\ldots }-{\bar{y}}_{\ldots l}+{\bar{y}}_{\ldots .}\right)}^{2}$	$\frac{{SS}_{AC}}{{DF}_{AC}}$	$\frac{{MS}_{AC}}{{MS}_{ErSP}}$
BC	(b − 1)(c − 1)	$\sigma_{sp}^{2}+\frac{ra\sum {\left(BC\right)}_{kl}^{2}}{\left(b-1)(c-1\right)}$	$ik\sum_{j,l}{\left({\bar{y}}_{.j.l}-{\bar{y}}_{.j..}-{\bar{y}}_{\ldots l}+{\bar{y}}_{\ldots .}\right)}^{2}$	$\frac{{SS}_{BC}}{{DF}_{BC}}$	$\frac{{MS}_{BC}}{{MS}_{ErSP}}$
ABC	(a − 1)(b − 1)(c − 1)	$\sigma_{sp}^{2}+\frac{r\sum {\left(ABC\right)}_{jkl}^{2}}{\left(a-1)(b-1)(c-1\right)}$	$k\sum_{i,j,l}{\left({\bar{y}}_{ij.l}+{\bar{y}}_{i\ldots }+{\bar{y}}_{.j..}+{\bar{y}}_{\ldots l}-{\bar{y}}_{ij..}-{\bar{y}}_{.j.l}-{\bar{y}}_{i..l}-{\bar{y}}_{\ldots .}\right)}^{2}$	$\frac{{SS}_{ABC}}{{DF}_{ABC}}$	$\frac{{MS}_{ABC}}{{MS}_{ErSP}}$
Error_SP_	ab(c − 1)(r − 1)	$\sigma_{sp}^{2}$	$\sum_{i,j,k,l}{\left(y_{ijkl}-{\bar{y}}_{ij.l}-{\bar{y}}_{ijk.}+{\bar{y}}_{ij..}\right)}^{2}$	$\frac{{SSE}_{ErSP}}{{DF}_{ErSP}}$	

**Table 4:** Analyzed ANOVA table for split-plot design.

Effect	DF	SS	MS	F_0_	*p*-Value
Temperature (A)	1	0.7979	0.7979	550.7	1.95 × 10^−5^ *
Heating Time (B)	1	0.3263	0.3263	225.3	1.15 × 10^−4^ *
AB	1	0.1733	0.1733	119.6	3.97 × 10^−4^ *
WP_Error_	4	0.0058	0.0015		
Compression Ratio (C)	1	0.2155	0.2155	627.1	1.51 × 10^−5^ *
AC	1	0.0794	0.0794	230.9	1.09 × 10^−4^ *
BC	1	0.0153	0.0153	44.56	2.62 × 10^−3^ *
ABC	1	0.0005	0.0005	1.314	0.3156
SP_Error_	4	0.0014	0.0003		
Total	15	1.615			

* indicates the effect is significant.

**Table 5:** Fixed effects coefficients for the coded mixed model.

Term	Coefficient	95% CI
Intercept	−0.015	−0.030, 0
Temperature (A)	−0.223	−0.238, −0.208
Time (B)	−0.143	−0.158, −0.128
AB	0.104	0.089, 0.119
Compression (C)	−0.116	−0.124, −0.108
AC	−0.070	−0.079, −0.062
BC	−0.031	−0.039, −0.022

**Table 6:** Alternative split-plot design for a literature auxetic foam manufacturing investigation.

Observational Unit	Whole Plot	Temperature	Time	Cooling	Radial Compression	Axial Compression
1	1	135	15	Water	1.58	1.17
2	1	135	15	Water	2.53	1.17
3	1	135	15	Water	1.58	3
4	1	135	15	Water	2.53	3
…	…	…	…	…	…	…
32	8	135	12	Water	1.58	3

## Data Availability

The original contributions presented in the study are included in the article, further inquiries can be directed to the corresponding author.
